# The efficacy of SMART Arm training early after stroke for stroke survivors with severe upper limb disability: a protocol for a randomised controlled trial

**DOI:** 10.1186/1471-2377-13-71

**Published:** 2013-07-02

**Authors:** Sandra G Brauer, Kathryn S Hayward, Richard G Carson, Andrew G Cresswell, Ruth N Barker

**Affiliations:** 1 Division of Physiotherapy, School of Health and Rehabilitation Sciences, The University of Queensland, Brisbane, QLD 4072, Australia; 2 Trinity College Institute of Neuroscience & School of Psychology, Trinity College Dublin, Dublin, Ireland; 3 School of Psychology, Queen’s University Belfast, Belfast, United Kingdom; 4 School of Human Movement Studies, The University of Queensland, Brisbane, QLD 4072, Australia; 5 Discipline of Physiotherapy, School of Public Health, Tropical Medicine & Rehabilitation Sciences, James Cook University Townsville, Townsville, Australia

**Keywords:** Stroke, Upper limb, Function, Training, Rehabilitation

## Abstract

**Background:**

Recovery of upper limb function after stroke is poor. The acute to subacute phase after stroke is the optimal time window to promote the recovery of upper limb function. The dose and content of training provided conventionally during this phase is however, unlikely to be adequate to drive functional recovery, especially in the presence of severe motor disability. The current study concerns an approach to address this shortcoming, through evaluation of the SMART Arm, a non-robotic device that enables intensive and repetitive practice of reaching by stroke survivors with severe upper limb disability, with the aim of improving upper limb function. The outcomes of SMART Arm training with or without outcome-triggered electrical stimulation (OT-stim) to augment movement and usual therapy will be compared to usual therapy alone.

**Methods/Design:**

A prospective, assessor-blinded parallel, three-group randomised controlled trial is being conducted. Seventy-five participants with a first-ever unilateral stroke less than 4 months previously, who present with severe arm disability (three or fewer out of a possible six points on the Motor Assessment Scale [MAS] Item 6), will be recruited from inpatient rehabilitation facilities. Participants will be randomly allocated to one of three dose-matched groups: SMART Arm training *with* OT-stim and usual therapy; SMART Arm training *without* OT-stim and usual therapy; or usual therapy alone. All participants will receive 20 hours of upper limb training over four weeks. Blinded assessors will conduct four assessments: pre intervention (0-weeks), post intervention (4-weeks), 26 weeks and 52 weeks follow-up. The primary outcome measure is MAS item 6. All analyses will be based on an intention-to-treat principle.

**Discussion:**

By enabling intensive and repetitive practice of a functional upper limb task during inpatient rehabilitation, SMART Arm training with or without OT-stim in combination with usual therapy, has the potential to improve recovery of upper limb function in those with severe motor disability. The immediate and long-term effects of SMART Arm training on upper limb impairment, activity and participation will be explored, in addition to the benefit of training with or without OT-stim to augment movement when compared to usual therapy alone.

**Trial registration:**

ACTRN12608000457347

## Background

Recovery of the upper limb after stroke is poor. Up to 80% of stroke survivors have some upper limb disability during the acute to subacute phase after stroke. By various estimates, only 5% to 20% demonstrate complete functional recovery [1-3]. Thus, stroke survivors with upper limb disability appear to be a rehabilitation challenge. There is therefore, a pressing need to increase the potential for functional recovery of the upper limb after stroke.

To drive recovery of function, it is recommended that training commence early and be intensive, repetitive and task-oriented [4-6]. However, stroke survivors with severe motor disability are often unable to participate in task-oriented training as they are incapable of generating levels of volitional motor activity or control that are sufficient to engage in training tasks [7]. Further compounding their situation is a lack of access to interventions that make task-oriented practice possible [8]. It is therefore not surprising that priority is rarely given to upper limb training by stroke survivors [9] or rehabilitation services [10-15] during inpatient rehabilitation. Yet, with stroke survivors spending up to 25% of a physiotherapy session inactive [15], there appears to be considerable scope within current therapeutic regimes to increase the delivery of task-oriented upper limb training.

**Figure 1:**
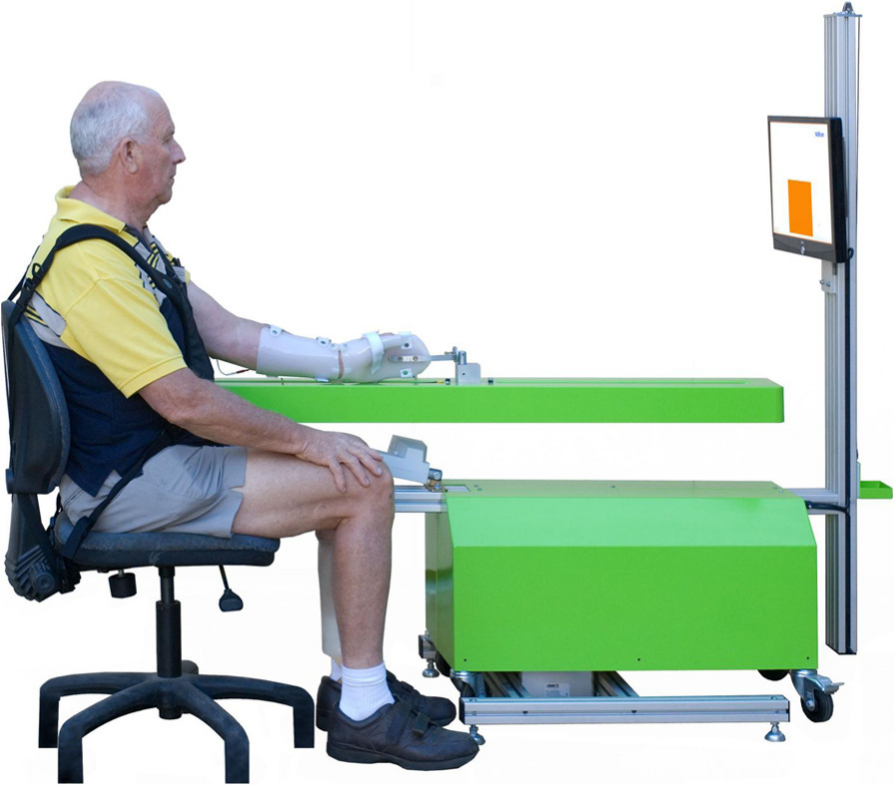
SMART Arm.

To capitalise upon this opportunity, a non-robotic training device, the SMART Arm (Sensori-Motor Active Rehabilitation Training of the Arm) (Figure 1), was developed to enable stroke survivors with severe upper limb disability to undertake intensive and repetitive task-oriented training. The device was specifically designed so that stroke survivors with little to no muscle activity could practice reaching, a fundamental upper limb function, along a straight-line path consistent with a normal reaching pattern. The device can be used with or without electrical stimulation to the lateral head of triceps brachii to augment elbow extension and enhance completion of the reaching task. To optimise the potential for motor learning, this device incorporates elements critical to skill acquisition, including active problem solving, augmented real-time feedback of performance, task progression that is tailored to each individual, motivation and encouragement.

To date, a 12-hour SMART Arm training program with or without electromyographic (EMG)-triggered electrical stimulation to triceps has been investigated in chronic stroke survivors with severe upper limb disability. Compared with the control group, those who underwent SMART Arm training with or without stimulation showed a significant improvement in upper limb function (improvement in MAS6 item 6 score) that was sustained at 2 months follow-up [16], an improved ratio of triceps to biceps EMG activity during reaching [17], and greater corticospinal reactivity [18]. There were however, variations in the expression of additional benefits derived from the use of EMG-triggered electrical stimulation. As this may have been due to the use, by some individuals, of maladaptive patterns of EMG activity such as co-contraction of biceps and triceps, that could nonetheless trigger stimulation, a new method of outcome-triggered electrical stimulation (OT-stim) was developed. Here, electrical stimulation is triggered when initial goal directed motion surpasses an individualised threshold. Thus, assistance by means of electrical stimulation (and reinforcement) occurs when the movement generated voluntarily is commensurate with the desired outcome. In a pilot trial of SMART Arm with OT-stim during inpatient rehabilitation, SMART Arm training (with or without OT-stim), led to a significantly greater improvement in upper limb function as compared to usual therapy alone [19]. In that these improvements were evident early after stroke, further investigation within the context of a larger trial is warranted [20].

Thus, the primary aim of the current randomised controlled trial (RCT) is to determine the ability of SMART Arm training with or without OT-stim compared to usual therapy to improve upper limb function in stroke survivors with severe upper limb disability undergoing inpatient rehabilitation. In addition, we will determine the impact of the different training programs on upper limb impairment, activity and participation.

## Methods/Design

### Design

**Figure 2:**
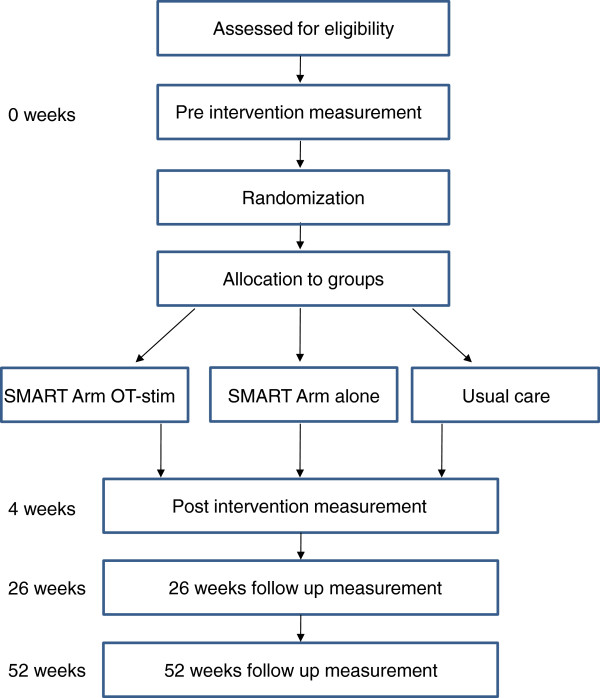
Trial design.

A prospective, assessor-blinded, three group parallel RCT will be conducted with 75 stroke survivors with severe upper limb disability who are undertaking inpatient rehabilitation (Figure 2).

### Location and setting

We plan to recruit stroke survivors from two inpatient rehabilitation services located in Brisbane, Australia: the Princess Alexandra Hospital, with a six-bed Acute Stroke Unit, located separately to a 78-bed Geriatric and Rehabilitation Unit; and the Queen Elizabeth II Jubilee Hospital, which has a four bed Acute Stroke Unit, co-located within a 24-bed Geriatric and Rehabilitation Unit. Assessment and training will be undertaken in different areas of the same site.

### Population

All stroke survivors admitted to the Acute Stroke Unit at each hospital will be screened for eligibility. Participants will be eligible if they are adult stroke survivors (>17 years) with a primary diagnosis of first-ever unilateral stroke (ischaemic or haemorrhagic, including subarachnoid haemorrhage) less than four months previously, which has been confirmed either radiographically (CT or MRI) or clinically by the consulting physician; demonstrate severe upper limb disability equivalent to a score of three or fewer out of a possible six points (inability to hold the upper limb in position when placed at 90° shoulder flexion in sitting) on the MAS item six; and are able to follow single-stage commands, either with verbal instructions, demonstration or other non-verbal cues.

Participants will be excluded if they are medically unstable as defined by the medical registrar or by location in an acute medical ward; have upper limb comorbidities that could limit their functional recovery (e.g., arthritis, pain, other neurological disorders); have a contraindication to the use of (e.g., pacemaker insitu), or inability to tolerate, electrical stimulation (e.g., hypersensitivity or skin condition); have an infectious disease requiring the use of personal protective equipment (e.g., methicillin resistant staphylococcus aureus or vancomycin resistant enterococcus) or are unable to sit without support.

### Randomisation and blinding

All participants will provide written informed consent. In the event that a participant is unable to provide informed consent, consent will be sought from their legal guardian. After completion of the initial assessment, participants will be randomised to one of three dose-matched groups (two intervention and one control). The intervention groups are SMART Arm training *with* OT-stim (SMART Arm OT-stim) and usual therapy; SMART Arm training *without* OT-stim (SMART Arm alone) and usual therapy. The control group will receive usual therapy only. Concealed randomisation will be prepared by an offsite investigator, not involved in recruitment, intervention or data collection, using a computer generated random number sequence. Consecutively numbered, randomly ordered opaque envelopes containing group allocation in permuted blocks of four or six in a 1:1 ratio will be opened consecutively after baseline assessment in the presence of the participant. Usual therapists will be informed of group allocation.

Research assistants who enrol participants, and conduct pre, post and follow-up assessments will be blinded to group allocation throughout the study. Participant coding will not refer to group allocation and participants will be instructed not to divulge information regarding their intervention to the assessors during assessment. Participants, SMART Arm trainers and usual therapists (physiotherapy and occupational therapy) will not be blinded to group allocation. To control expectancy effects for participants and usual therapists, it will be explained that it is not yet determined which therapy is more effective.

### Intervention

All participants will receive 20 hours of upper limb therapy, comprising 60 minutes duration five days per week for four weeks. The proposed volume of training was guided by discussions with each site and reports from these [15] and other facilities [21], along with previous SMART Arm research [16,19]. All SMART Arm training and usual therapy will be recorded in individual participant logbooks. If a participant misses a SMART Arm or usual therapy session due to illness, medical procedure, or extended leave (e.g., returned to acute medical ward as became unstable), additional days will be added to ensure all participants are given the opportunity to complete a total of 20-days of therapy.

#### SMART Arm training

SMART Arm training will be administered for 30 minutes per day by a physiotherapist or occupational therapist, trained in the delivery of the intervention. The participant’s treating therapy team will administer 30 minutes of usual therapy per day. Training will be typically undertaken five times per week for four weeks.

On commencement of a SMART Arm training session, the participant will be seated on a (armless) chair beside the device. A harness will be applied to restrict trunk movements to less than 15 degrees and therefore, minimise compensatory trunk movements and encourage recovery of a pre-morbid pattern of reaching [22-24]. The affected upper limb will be positioned in a customised thermoplastic splint in mid pronation-supination and wrist extension (0 degrees to 45 degrees) to mimic a functional reach-to-grasp hand position [25]. To accommodate for any muscle contracture or pain, the splint can be positioned through the full range of pronation and supination. The splint is connected to a manipulandum, which is mounted on a linear slide and encoder belt. The linear slide serves to constrain movement to one plane and to reduce friction and resistance to movement. The elbow is positioned in a standardized start position of 90 degrees of elbow flexion.

Trainers will be provided with guidelines for the administration of SMART Arm training. To ensure participants perform a consistent minimum number of repetitions during the training time period (30 minutes), a goal of a minimum of 60 repetitions in week one and 80 repetitions in weeks two through four will be set. This dose was guided by previous research [16,19]. Progression in training difficulty will occur when consistency in task practice is evident. Training elements that can be progressed include the number of repetitions, track elevation, degree of shoulder external rotation, hand position, load, visual and auditory feedback, instruction and level of supervision. The decision-making process with regards to when and how to progress training will be at the discretion of the SMART Arm trainer and will be based on the stroke survivor’s performance during training. To ensure consistency between trainers, monitoring (e.g., benchmarking evaluations of completed training logs for consistent dose, progressions of practice used) and mentoring (e.g., peer-supervision, feedback during sessions, and debriefing) will regularly occur. All SMART Arm training will be documented in a log, which will capture dose and training element use.

##### Outcome-triggered electrical stimulation

The lateral head of triceps brachii is the target muscle of electrical stimulation as it is the prime mover for achievement of full elbow extension. Stimulation to triceps brachii will be delivered via an Empi 300PV unit (St Paul, MN, USA). Two surface electrodes (diameter 50 mm) will be applied, one above the area of the triceps brachii motor point (lateral head) and one at the muscle insertion. Stimulation parameters will consist of a 1 second ascending ramp, and a 4 to 20 second duration of 200-sec pulse width biphasic stimulation at 35 Hz. When training is commenced, the participant will attempt to initiate the reaching task. As the participants’ reach attempt surpasses their individually determined threshold distance, electrical stimulation to triceps brachii will be automatically triggered. The duration of stimulation provided will be set manually to match the time required by each participant to perform the movement. The stimulus intensity will be set to the maximum that can be tolerated by the participant.

#### Usual therapy

Participants allocated to usual therapy alone will participate in 20 therapy sessions of 60 minutes duration, typically undertaken five days per week for four weeks. Usual therapy refers to the combined duration of occupational therapy and physiotherapy. It will not be standardized and will likely consist of a mix of individual and group sessions administering both passive (e.g. stretching, cyclic electrical stimulation) and active (e.g. range of movement, strengthening, modified task practice with electrical stimulation) interventions where possible. All usual therapy will be documented in an upper limb therapy log, which will capture dose (minutes), frequency (sessions) and content of upper limb therapy.

### Outcome measures

Arm function (impairment, activity and participation) will be assessed in accordance with the ICF Classification of Functioning, Disability and Health [26]. All participants will be assessed at four time points: three days prior to commencement of the intervention (baseline, 0 weeks), within one week of completion of the intervention (post-intervention, 4 weeks), and following completion of the intervention at 26 and 52 weeks. Assessors will be provided with guidelines for administering the measures.

Demographic information about participants will be collected from their medical record and will include age, gender, date of stroke onset, type (ischaemic or haemorrhagic) and location (cortical, subcortical, cortical and subcortical or brainstem) of stroke, stroke medical intervention (e.g. thrombolysis), co-morbidities and medications.

#### Primary outcome measure

The primary outcome measure will be performance on the MAS item 6 at the post intervention time period (4 weeks). The MAS is designed to measure recovery of the affected limb over 3 task-related subscales (upper arm function, hand and advanced hand movements) that are scored from 0–6. It is the stroke recovery scale most commonly used in clinical practice in Australia and takes less than 10 minutes to complete. The reliability and validity of this measure with the stroke population has been previously documented [27]. It has been shown to be sensitive to change in performance in people with severe upper limb disability after training with SMART Arm [16,19].

#### Secondary laboratory outcome measures

The functional force generating capacity of the impaired limb will be assessed using a dynamic and an isometric reaching task similar to previous protocols [16,17]. In both tasks, surface EMG activity will be collected from triceps brachii lateral head, biceps brachii, anterior deltoid, upper trapezius, external rotators, lower trapezius and serratus anterior. EMG will be obtained using single differential pre-amplified (gain 1000) parallel bar electrodes (Bagnoli, DELSYS, 8-channel System, Boston, MA, USA) with a fixed inter-electrode distance of 10 mm and positioned according to SENIAM guidelines [28]. A reference electrode will be attached over the bony prominence of the seventh cervical spinous process. Signals will be sampled (1000Hz) using a Power 1401 Data Acquisition System (Cambridge Electronics Design, Cambridge, UK) and Spike2 software (version 6.02). Time series data will be collected and stored using Spike2 and processed using custom routines in Matlab (Mathworks Inc., Nattick, MA).

Participants will be seated at a custom-built apparatus with the upper limb in a pendant position, the elbow flexed and the forearm and hand restrained in pronation via a custom built brace. For all measures a computer monitor positioned directly in front of the participant will provide visual feedback on a vertical bar scale. In the dynamic task, the brace will be secured to a manipulandum mounted on a linear slide restricting motion of the upper limb to flexion/ extension of the shoulder and elbow. A potentiometer attached to the slide will measure transducer, reaching, linear displacement. The upper limb will be placed at a standardised starting position with the elbow at 90 degrees of flexion. Upon presentation of a tone, participants will be required to ‘reach forward as far as possible’ in five separate trials. In the isometric task, the participant will be required to push forwards (elbow extension) in a position of 150 degrees of elbow flexion against a manipulandum instrumented to measure force. In five separate trials, following a tone, participants will be instructed to ‘push as hard as possible’ and to keep pushing for five seconds. In both tasks continuous visual feedback of the applied force will be provided along with verbal encouragement. During each contraction, force or reach distance and EMG recordings will be obtained. On the basis of these recordings, peak force, distance, time to peak and the muscle onset times, amplitude (root-mean-square (RMS)) and triceps to biceps ratio of EMG RMS amplitude will be calculated.

In a subset of the participants, the collection of EMG signals will be triggered and synchronized using an OptiTrack™ 6 camera 120 Hz system, with Tracking Tools™ computer software (NaturalPoint, Inc, OR, USA) which will collect kinematic data. Upper limb movement will be tracked via the recording of reflective marker clusters placed on the participants’ upper arm, distal forearm, sternum, and acromion and acromioclavicular joints. Kinematic data for analysis will include displacement, velocity and changes in angles of upper arm segments and markers.

#### Secondary outcome measures: clinical

A range of clinical measures will be collected to measure the presence of impairments post stroke. To measure strength, lateral head of triceps brachii muscle power will be assessed using manual muscle testing according to MRC ratings from 0–5 [29]. To measure the active range of movement of finger flexion and extension, thumb extension and abduction, elbow flexion and extension and shoulder abduction and adduction, assessment according to the protocol described by Uswatte et al. [30] will be performed. To measure the presence of spasticity of biceps brachii and resistance to passive elbow extension, the modified Ashworth Scale [31] and Tardieu Scale [32] will be administered. To evaluate joint tenderness on passive movement of the hemiplegic shoulder, the Ritchie Articular Index will be administered [33,34]. To describe participants at baseline only, the Cognitive Linguistic Quick Test [35] and Nottingham Sensory Assessment [36,37] will be administered. Motor Assessment Scale items 7 (hand movements) and 8 (advanced hand activities) will be performed to monitor for any carryover improvement in hand function. To examine upper limb function at the participation level, two self-report measures will be used. The Stroke Impact Scale will be used to measure the impact of the intervention on the stroke survivor’s recovery [38]. The Motor Activity Log will be administered to all subjects to rate how well and how much they use the paretic limb spontaneously in everyday tasks [30].

### Data analysis

Data analysis will be performed on an intention-to-treat basis using an alpha level of 0.05. Descriptive statistics will be used to ensure comparability of scores between groups at baseline, to describe performance at each phase and to test whether the assumptions for use of parametric statistics have been met. If the assumptions for F or t-tests are violated, equivalent non-parametric statistics will be utilized. The main hypothesis will be tested using mixed effects models, in a 3 group (SMART Arm + OT stim, SMART Arm alone, usual therapy) × 4 phase (0, 4, 26, 52 weeks) model. This will be followed by between-groups planned comparisons. All secondary outcomes will be analysed in a similar manner.

### Sample size

The principle endpoint is post intervention (4 weeks). Our previous RCT demonstrated that stroke survivors using the SMART Arm alone or with electrical stimulation demonstrated significant improvements in MAS-6 scores compared to the control group [16]. On this basis, we estimate a mean improvement of 1.8 (SD 2) in the usual therapy group, 2.91 (SD 2) in the SMART Arm alone group and 3.91 (SD 2) in the SMART Arm with OT-stim group. To achieve 80% power and a significance of 0.05 with pairwise comparisons, 22 subjects are required per group, to total 66 subjects. A 15% dropout rate will be allowed to account for withdrawals, thus 25 subjects will be recruited per group, totalling 75.

### Ethics approval

The study protocol has been approved by the University of Queensland Medical Research Ethics Committee (MREC ID: 2007001628), and the Princess Alexandra Hospital Ethics Committee (ID: 2008–046). This study will be conducted in accordance with the Declaration of Helsinki.

## Discussion

This will be the first prospective trial to compare the effect of dose-matched volumes of SMART Arm with OT-stim and usual therapy, versus SMART Arm alone with usual therapy versus usual therapy alone during inpatient rehabilitation following stroke. While it is known that intensive and repetitive, task-oriented training is critical to drive motor recovery after stroke [39,40], those with severe impairment and ‘not enough movement to work with’ require assistance to complete a functional movement pattern. Currently, the most commonly used method is manual assistance by a therapist. The minimal time spent on the upper limb during physiotherapy indicates however, that this time-inefficient strategy is not prioritised. It is likely that gait training requirements, which are paramount to determining discharge destination, are prioritised. Another option is robotic therapy, however availability is currently limited and functional outcomes remain inconclusive [8,41,42].

In the event that SMART Arm training, with or without electrical stimulation, leads to demonstratable significant improvements in upper limb function, reduced impairments or increased participation, an alternative option for retraining would be presented. Improvements in impairments and activity using the SMART Arm have been obtained in chronic stroke survivors [16], and the indications from our pilot work are that similar outcomes may be achieved in those undertaking inpatient rehabilitation [19]. If positive changes may be induced during inpatient rehabilitation, this may allow some stroke survivors to regain levels of function that are sufficient to enable progression to interventions such as constraint induced movement therapy.

This study will also allow the impact of augmenting training with electrical stimulation to be assessed in the context of severe motor disability. This will be the first large study to determine the effect of outcome-triggered stimulation, in circumstances in which the stroke survivor is *rewarded* for performing the desired movement, rather than for simply generating sufficient levels of EMG i.e. regardless of the functional consequences.

Findings from this study will provide insights into the effects of practice on regaining motor skill in those with severe upper limb disability following stroke. The collection of detailed training data will generate new knowledge regarding the importance of specific training elements, such as load and feedback on performance in the early phase of rehabilitation. These will have implications that extend beyond the current modes of training investigated and possibly to other populations such as those with other forms of brain injury.

## Consent

Written informed consent was obtained from the patient for publication of the image (Figure 1). A copy of the written consent is available for review by the Editor of this journal.

## Abbreviations

ANCOVA: Analysis of covariance; EMG: Electromyography; MAS: Motor assessment scale; RCT: Randomised controlled trial; RMS: Root mean square; SENIAM: Surface electromyography for the non-invasive assessment of muscles; SPSS: Statistical processes for the social sciences.

## Competing interests

SG Brauer, KS Hayward, RN Barker and RG Carson are currently involved in commercialisation of the SMART Arm device.

## Authors’ contributions

SB, RB, and RC conceived the idea for the study. SB, RB, RC and AC all contributed to the research design and obtained funding for the study. SB, KH, RB, RC and AC contributed to the design of the study, intervention and outcome measures. SB, KH and RB were involved in participant recruitment. SB and KH were principally responsible for the drafting of the manuscript. All authors assisted in editing the final submitted manuscript. All authors read and approved the final manuscript.

## Pre-publication history

The pre-publication history for this paper can be accessed here:

http://www.biomedcentral.com/1471-2377/13/71/prepub
